# Association of Vascular Endothelial Growth Factor A-2549 I/D polymorphism with polycystic ovary syndrome susceptibility in Turkish women

**DOI:** 10.1590/1806-9282.20260842

**Published:** 2026-10-16

**Authors:** Recai Aci, Mehmet Can Nacar, Serbulent Yigit, Husniye Rustemoglu, Eda Nacar

**Affiliations:** 1 Aydin Adnan Menderes University, Soke Vocational School of Health Services – Aydın, Turkey.; 2 Adiyaman University, Faculty of Medicine, Department of Obstetrics and Gynecology – Adıyaman, Turkey.; 3 Ondokuz Mayis University, Faculty of Veterinary Medicine, Department of Genetics – Samsun, Turkey.; 4 Aksaray University – Aksaray, Turkey.; 5 Adiyaman University, Faculty of Medicine, Department of Internal Medicine – Adıyaman, Turkey.

**Keywords:** Polycystic ovary syndrome, Vascular Endothelial Growth Factor A, Polymorphism, genetic, Insulin resistance, Genotype

## Abstract

**OBJECTIVE:**

The aim of this case-control study was to evaluate whether the Vascular Endothelial Growth Factor A promoter rs35569394 (-2549 insertion/deletion) polymorphism is associated with polycystic ovary syndrome risk and related clinical-metabolic features.

**METHODS:**

The study included 104 women with polycystic ovary syndrome diagnosed according to Rotterdam criteria and 128 controls. Clinical, hormonal, and metabolic variables were recorded, homeostatic model assessment of insulin resistance was calculated, and Vascular Endothelial Growth Factor A-2549 insertion/deletion genotypes were determined by direct polymerase chain reaction. Hardy-Weinberg equilibrium was assessed in controls. Benjamini-Hochberg false-discovery-rate-adjusted p-values were calculated for the 11 clinical comparisons, while prespecified Bonferroni-adjusted p-values were retained for genotype and family-history contrasts.

**RESULTS:**

Body mass index, prolactin, homeostatic model assessment of insulin resistance, insulin, follicle stimulating hormone, luteinizing harmone, and total testosterone were higher in the polycystic ovary syndrome group. Overall genotype distribution differed between groups (Cramer's V=0.20; chi-square=8.87, p=0.012). The detection/deletion genotype was more frequent in polycystic ovary syndrome patients than controls (30.77% vs. 15.63%; Cohen's h=0.36; raw p=0.007; Bonferroni-adjusted p=0.028; OR 2.40, 95%CI 1.28–4.51). The family-history detection/deletion comparison did not remain significant after adjustment (raw p=0.018; adjusted p=0.072). Hardy-Weinberg equilibrium analysis in controls yielded an exact p-value of 0.050 and a Pearson p-value of 0.037.

**CONCLUSION:**

In this Turkish case-control sample, the Vascular Endothelial Growth Factor A-2549 insertion/deletion detection/deletion genotype was more frequent among women with polycystic ovary syndrome than among controls. This observed association does not establish causality, a functional effect, phenotype-specific relevance, or clinical utility. Deviation from Hardy-Weinberg equilibrium in controls, residual confounding from incomplete matching, limited power, and lack of functional validation make the finding preliminary. Independent validation in larger, well-matched cohorts with prespecified genotyping quality control, phenotype stratification, and functional studies is required.

## INTRODUCTION

Polycystic ovary syndrome (PCOS) is a common endocrine-metabolic disorder characterized by ovulatory dysfunction, hyperandrogenism and/or polycystic ovarian morphology^1,2^. Its heterogeneous expression is influenced by adiposity, insulin resistance, gonadotropin imbalance and genetic background. Prevalence varies by diagnostic criteria and ethnicity, supporting the study of population-specific genetic markers^3–5^. The contribution of angiogenesis-related variants remains incompletely defined.

The promoter variant rs35569394 (-2549 insertion/deletion (I/D)) is an 18-bp insertion/deletion located in the VEGFA promoter and has been hypothesized to influence transcriptional regulation; however, its functional direction and magnitude in ovarian tissue remain uncertain.

Associations between VEGFA polymorphisms and PCOS vary across populations, possibly because of ethnic, linkage-disequilibrium and environmental differences^6–13^. Evidence regarding rs35569394 in PCOS remains limited and population dependent, and the variant has not been adequately characterized in Turkish women with PCOS. Familial aggregation makes family history a clinically relevant, although imprecise, marker of inherited susceptibility. We therefore hypothesized that rs35569394 might be associated with PCOS status and explored whether genotype patterns differed according to self-reported family history and metabolic features. Any positive result was considered hypothesis-generating rather than immediately clinically actionable.

## METHODS

### Study population and design

This prospective case-control study was conducted at the Department of Obstetrics and Gynecology, Faculty of Medicine, Adiyaman University. The case group included 104 women diagnosed with PCOS according to the Rotterdam criteria, and the control group included 128 healthy women with regular menstrual cycles and no clinical, biochemical or ultrasonographic evidence of PCOS. Controls were recruited pragmatically from eligible women available during the study period. Individual matching, a fixed matching ratio, and matching calipers were not prespecified. Recruitment aimed to approximate the age and body mass index (BMI) distributions at group level, but BMI remained higher in the PCOS group. Written informed consent was obtained from all participants. The study was approved by the Adiyaman University Ethics Committee (decision date: 24 May 2022; meeting no. 5; decision no. 2022/5/19) and conducted in accordance with the Declaration of Helsinki.

### Sample size and power analysis

An a priori sample-size calculation was performed using G*Power software (version 3.1.9.4). For a chi-square test comparing the 2×3 genotype distribution between the PCOS and control groups, an effect size of Cohen's w=0.215 was assumed based on previously reported rs35569394 genotype distributions in PCOS^6^. With alpha=0.05 and 80% statistical power, the minimum required total sample size was 209. Allowing for approximately 10% incomplete or unusable data, the recruitment target was set at 230 participants. The final sample comprised 232 women (104 with PCOS and 128 controls), exceeding the planned total. Subgroup and multiplicity-adjusted analyses nevertheless had lower effective power and were interpreted cautiously.

### Eligibility and family history

PCOS was defined by the presence of at least two Rotterdam criteria: oligo-anovulation, clinical and/or biochemical hyperandrogenism, and polycystic ovarian morphology. Controls had regular 21–35-day cycles, normal ovarian morphology, and no evidence of hyperandrogenism. Although all cases met Rotterdam criteria, the four component-defined phenotypes were not recorded in a form permitting reliable retrospective A–D classification. The pooled analysis may therefore conceal phenotype-specific genetic and metabolic associations. Exclusion criteria were pregnancy, lactation, hormonal contraceptive or insulin-sensitizing drug use within the preceding 3 months, hyperprolactinemia, thyroid dysfunction, Cushing syndrome, congenital adrenal hyperplasia, chronic systemic disease, hepatic or renal failure and malignancy.

A positive family history was defined as a previous diagnosis of PCOS in the participant's mother or sister. Family-history information was obtained through structured personal interviews and was not independently verified from relatives’ medical records. Family-history analyses were therefore considered exploratory and susceptible to recall and classification bias. Controls were not excluded based on a family history of PCOS, as routine verification of relatives’ diagnoses was not feasible; this is acknowledged as a limitation that may attenuate the genetic contrast between groups.

### Clinical and biochemical assessment

Fasting venous blood samples were collected in the early follicular phase (cycle days 2–5). Serum was separated by centrifugation and stored at −80°C until analysis. Follicle stimulating hormone (FSH) (Elecsys FSH), LH (Elecsys LH), estradiol (Elecsys Estradiol III), prolactin (Elecsys Prolactin II), thyroid-stimulating hormone (TSH) (Elecsys TSH), total testosterone (Elecsys Testosterone II), and fasting insulin (Elecsys Insulin) were measured by electrochemiluminescence immunoassay on a Roche cobas e801 analyzer (Roche Diagnostics, Mannheim, Germany).

Manufacturer-stated analytical measuring ranges (LoD; LoQ) were as follows: FSH, 0.3–200 mIU/mL (0.3; 1.0 mIU/mL); LH, 0.3–200 mIU/mL (0.3; 1.0 mIU/mL); estradiol, 5–3,000 pg/mL (5; 25 pg/mL); prolactin, 0.094–470 ng/mL (0.094; 0.940 ng/mL); TSH, 0.005–100 μIU/mL (0.005; 0.005 μIU/mL); insulin, 0.4–1,000 μU/mL (0.4; 1.0 μU/mL); and total testosterone, 0.087–52.0 nmol/L (0.087; 0.416 nmol/L). Representative manufacturer precision [repeatability/intermediate-precision coefficient of variation (CV)] ranges were FSH, 0.8–2.1%/3.2–3.8%; LH, 0.9–2.2%/1.1–2.3%; estradiol, 1.2–8.4%/1.8–12.3%; prolactin, 1.8–3.1%/2.7–3.8%; TSH, 0.7–3.4%/1.8–11.2%; insulin, 0.8–4.3%/1.4–5.3%; and total testosterone, 1.8–11.1%/3.5–16.8%, depending on concentration.

Corresponding manufacturer reference intervals used for contextual interpretation were FSH 3.5–12.5 mIU/mL and LH 2.4–12.6 mIU/mL in the follicular phase, estradiol 20.5–62.8 pg/mL in the early follicular phase, prolactin 4.79–23.3 ng/mL in nonpregnant women, TSH 0.270–4.20 uIU/mL, fasting insulin 2.6–24.9 μU/mL, and total testosterone 0.291–1.668 nmol/L in apparently healthy women aged 20–49 years. Total testosterone values were converted from ng/mL to nmol/L using 1 ng/mL=3.467 nmol/L for reporting. Fasting glucose was measured by the hexokinase method on a Beckman Coulter AU5800 analyzer. Insulin resistance was estimated using HOMA-IR: fasting insulin (μU/mL)×fasting glucose (mg/dL)/405^14^.

### Genetic analysis

Genomic DNA was isolated from ethylenediaminetetraacetic acid (EDTA)-anticoagulated peripheral blood using a DNeasy kit (Qiagen, Hilden, Germany) according to the manufacturer's instructions. DNA concentration and purity were assessed by NanoDrop spectrophotometry, and samples with adequate A260/A280 ratios were used for genotyping. The 18-bp insertion/deletion polymorphism at position −2549 in the VEGFA promoter (rs35569394) was genotyped by direct polymerase chain reaction (PCR), based on the previously published Turkish VEGFA protocol^15^. The forward primer was 5′-GCTGAGAGTGGGGCTGACTAGGTA-3′ and the reverse primer was 5′-GTTTCTGACCTGGCTATTTCCAGG-3′. PCR was performed in a final volume of 25 μL containing approximately 100 ng genomic DNA, 1×PCR buffer, 2.0 mM MgCl2, 0.2 mM of each dNTP, 20 pmol of each primer, and 1 U Taq DNA polymerase. Amplification consisted of initial denaturation at 95°C for 10 min; 35 cycles at 95°C for 45 s, 60°C for 45 s, and 72°C for 45 s; and final extension at 72°C for 10 min. PCR products were separated on a 2.5% agarose gel and visualized using a Bio-Rad ChemiDoc system. The 229-bp fragment represented the insertion allele and the 211-bp fragment represented the deletion allele; II, detection/deletion (DD), and ID genotypes were identified by the 229-bp band, the 211-bp band, or both bands, respectively. To assess reproducibility, 20% of randomly selected samples were reanalysed, with complete genotype concordance.

### Statistical analysis

Analyses were performed using International Business Machines Statistical Package for the Social Sciences (IBM SPSS Statistics), version 26.0 (IBM Corp., Armonk, NY, USA). Normality was evaluated using the Shapiro-Wilk test. Continuous variables are presented as mean +/- standard deviation and categorical variables as n (%). Group comparisons used independent-samples t-tests or chi-square tests, as appropriate. The overall 2×3 genotype distribution was evaluated before genotype-specific comparisons. Genotype and allele distributions were compared between groups, and odds ratios (ORs) with 95%CIs were calculated. Effect sizes were summarized using Cramer's V for the overall 2×3 genotype comparison and Cohen's h for the DD genotype comparison. HWE in controls was evaluated using both the asymptotic Pearson chi-square test and an exact test because results near the conventional 0.05 threshold can differ owing to the discrete genotype distribution and the approximation used by the Pearson procedure. Benjamini-Hochberg false-discovery-rate (FDR)-adjusted p-values are reported for the 11 clinical comparisons. The prespecified Bonferroni-adjusted values are retained for genotype and family-history contrasts. A single global correction across all clinical, genotype, and family-history tests was not performed because the a priori statistical plan specified domain-based corrections (FDR for clinical variables, Bonferroni for genetic and family-history contrasts) to control error rates within distinct biological families of tests. Because individual-level data were not retained for post hoc revision, a BMI-adjusted logistic regression model could not be reconstructed, which is acknowledged as a limitation. Although the final sample exceeded the a priori recruitment target, negative subgroup and multiplicity-adjusted findings may still reflect limited power.

## RESULTS

Clinical and biochemical comparisons are summarized in Table 1. After Benjamini-Hochberg FDR correction for 11 clinical comparisons, BMI, prolactin, HOMA-IR, insulin, FSH, LH and total testosterone remained statistically higher in the PCOS group than in controls (all FDR-adjusted p≤0.005). Total testosterone was 3.16 +/- 0.45 nmol/L in women with PCOS and 2.08 +/- 1.25 nmol/L in controls (raw p<0.001; FDR-adjusted p=0.002). Glucose, estradiol, TSH, and age at menarche did not differ significantly after FDR correction (FDR-adjusted p=0.165, 0.219, 0.113, and 0.627, respectively).

**Table 1:** Clinical and laboratory findings.

Characteristics	Patients (n=104), mean +/- SD	Controls (n=128), mean +/- SD	Raw p	FDR-adjusted p
BMI (kg/m^2^)	27.325±3.587	25.23±4.271	<0.001	0.002
Age at menarche (year)	12.27±0.601	12.36±1.72	0.627	0.627
Prolactin (ng/mL)	13.4±8.934	9.63±6.06	0.003	0.005
HOMA-IR	2.3±2.13	1.55±1.6	0.001	0.004
Glucose (mg/dL)	85.2±12.63	87.63±9.31	0.135	0.165
Insulin (μU/mL)	10.5±6.13	7.67±6.06	0.001	0.004
E2 (Estradiol) (pg/mL)	78.95±62.38	90.31±71.23	0.199	0.219
FSH (mIU/mL)	9.31±3.65	7.85±3.41	0.003	0.005
LH (mIU/mL)	10.27±9.36	7.6±5.35	0.002	0.004
TSH (mIU/L)	1.80±2.13	1.23±3.62	0.082	0.113
TT (nmol/L)	3.16±0.45	2.08±1.25	<0.001	0.002

Values are presented as mean +/- standard deviation. Between-group comparisons used independent-samples t-tests. FDR-adjusted p-values were calculated by the Benjamini-Hochberg procedure for 11 clinical comparisons. Total testosterone is reported in nmol/L after conversion from ng/mL using 1 ng/mL=3.467 nmol/L. BMI: body mass index; HOMA-IR: homeostatic model assessment of insulin resistance; FSH: follicle-stimulating hormone; LH: luteinizing hormone; TSH: thyroid-stimulating hormone; E2: estradiol; TT: total testosterone; SD: standard deviation; FDR: false-discovery-rate.

The overall genotype distribution differed between groups (Cramer's V=0.20; chi-square=8.87, df=2, p=0.012). The DD genotype was more frequent among PCOS patients (30.77 vs. 15.63%; Cohen's h=0.36; raw p=0.007; adjusted p=0.028; OR 2.40, 95%CI 1.28–4.51) and was the only genotype-specific comparison that remained significant after Bonferroni correction. The lower ID frequency was nominally significant but did not remain significant after correction (raw p=0.017; adjusted p=0.068). The II genotype and D-allele comparisons were not significant (adjusted p=1.000 and 0.456, respectively). The genotype distribution in controls was at the HWE boundary by the exact test and below it by the Pearson test (DD/ID/II=20/75/33; Pearson chi-square=4.34, p=0.037; exact p=0.050) (Table 2).

**Table 2:** Genotype and allele frequencies of the Vascular Endothelial Growth Factor A-2549 I/D polymorphism.

Genotype/allele	Patients n	Patients %	Controls n	Controls %	Chi-square	Raw p	Adjusted p	OR (95%CI)
DD	32	30.77	20	15.63	7.241	0.007	0.028	2.40 (1.28–4.51)
ID	44	42.31	75	58.59	5.650	0.017	0.068	0.52 (0.31–0.87)
II	28	26.92	33	25.78	0.022	0.881	1.000	1.06 (0.59–1.90)
Total (genotype)	104	100	128	100				
D allele	108	51.92	115	44.92	2.510	0.114	0.456	1.35 (0.94–1.95)
I allele	100	48.08	141	55.08				
Total (allele)	208	100	256	100				

Overall genotype comparison: chi-square=8.87, df=2, p=0.012. Adjusted p-values use Bonferroni correction for four genotype/allele contrasts. Control HWE: Pearson chi-square=4.34, p=0.037; exact p=0.050. OR: odds ratio; CI: confidence interval.

Family-history subgroup findings are shown in Table 3. No genotype or allele comparison remained significant after Bonferroni correction. The DD comparison between familial PCOS and controls was nominally significant (raw p=0.018) but not after adjustment (p=0.072).

**Table 3:** Vascular Endothelial Growth Factor A-2549 I/D genotype and allele frequencies according to family history.

Genotype/allele	Family history (+), n=46	Family history (-), n=58	Controls, n=128	P1 raw (adjusted)	OR (95%CI)	P2 raw (adjusted)	OR (95%CI)
DD	15	17	20	0.831 (1.000)	1.17 (0.51–2.67)	0.018 (0.072)	2.61 (1.21–5.66)
ID	21	23	75	0.556 (1.000)	1.28 (0.59–2.78)	0.167 (0.668)	0.59 (0.30–1.16)
II	10	18	33	0.374 (1.000)	0.62 (0.25–1.50)	0.692 (1.000)	0.80 (0.36–1.78)
Total (genotype)	46	58	128				
D allele	51	57	115				
I allele	41	59	141	0.403 (1.000)	1.29 (0.75–2.22)	0.090 (0.360)	1.53 (0.95–2.46)
Total (allele)	108	100	256				

P1 compares PCOS patients with and without self-reported family history; P2 compares PCOS patients with positive family history and controls. Values in parentheses are Bonferroni-adjusted p-values across four contrasts. OR: odds ratio; CI: confidence interval.

## DISCUSSION

In this Turkish case-control cohort, the DD genotype of VEGFA −2549 I/D was more frequent among women with PCOS than among controls, whereas the allele-level comparison was not significant. The overall genotype distribution also differed between groups. These findings support a possible genotype-specific association but do not establish causality. Because rs35569394 is located in the VEGFA promoter, it has been hypothesized to modify transcriptional regulation; however, VEGFA expression, circulating protein concentration, ovarian tissue activity, and promoter function were not measured in this study. The mechanistic interpretation therefore remains biologically plausible but unconfirmed.

The clinical profile of the PCOS group was characterized by higher BMI, insulin, HOMA-IR, LH, and total testosterone, consistent with the endocrine-metabolic heterogeneity of PCOS^16–20^. Hyperinsulinemia may intensify ovarian androgen production and reduce hepatic sex hormone-binding globulin (SHBG), while adiposity can further modify gonadotropin and metabolic patterns. Total testosterone was higher in PCOS than in controls (3.16 +/- 0.45 vs. 2.08 +/- 1.25 nmol/L; FDR-adjusted p=0.002), supporting a group-level hyperandrogenic difference. Although the mean total testosterone in the control group slightly exceeded the manufacturer's general reference interval, it was significantly lower than in the PCOS group and remained within the expected physiological spectrum for reproductive-aged women in our laboratory setting. FSH was also modestly higher in cases. Because increased FSH is not a defining PCOS feature, this observation may reflect phenotype composition, cycle timing, assay characteristics, adiposity, or sampling variation^21^. The absence of free testosterone, SHBG, repeated measurements, and phenotype-specific hormonal profiles limits more detailed endocrine interpretation.

VEGFA is biologically relevant to PCOS because follicular development, ovulation, and corpus luteum formation depend on tightly regulated angiogenesis. Excessive or mistimed VEGFA signalling could contribute to increased stromal vascularity, vascular permeability, altered follicular maturation, and an exaggerated ovarian response. Contemporary granulosa-cell and ovarian-stimulation studies show that angiogenesis-related gene expression and VEGF-mediated vascular responses vary according to cellular and treatment context^22,23^. These observations support the ovarian relevance of the pathway but do not demonstrate that rs35569394 produces the same effects in PCOS. Associations involving circulating VEGF or other VEGFA variants have also varied among populations^6–12^, suggesting that ancestry, linkage disequilibrium, clinical phenotype, adiposity, and analytical methods may influence observed genetic effects.

The control-group HWE deviation remains an important methodological limitation. The exact test was borderline (p=0.050), whereas the Pearson test was below 0.05 (p=0.037). Complete concordance in the 20% repeat-genotyping subset reduces concern about random technical error but does not exclude systematic misclassification, population stratification, cryptic relatedness, or selection effects. The control genotype distribution may therefore not represent the underlying population frequencies, and the observed odds ratio should be interpreted cautiously.

The D-allele frequency was 51.9% among cases and 44.9% among controls in the present cohort. By comparison, Fernandes et al. reported a D-allele frequency of 0.64 in both Brazilian cases and controls and found no association with PCOS^7^. Their control group also departed from HWE. Differences in allele distribution and genotype structure between the Brazilian and Turkish cohorts illustrate the importance of ancestry, recruitment, and sampling framework in interpreting rs35569394. The present DD association, despite a non-significant allele comparison, may be compatible with a recessive or genotype-specific pattern; alternatively, it may result from limited power, control selection, residual confounding, or HWE deviation. Accordingly, replication should test prespecified dominant, recessive, and additive genetic models rather than focusing only on isolated genotype contrasts.

An important interpretive issue is residual confounding by adiposity. Although the groups were broadly similar in age, BMI was significantly higher in the PCOS group. BMI may influence both PCOS status and metabolic or angiogenic phenotypes; therefore, the observed DD association cannot be interpreted as independent of adiposity because an individual-level covariate-adjusted model was not available for revision. Future studies should use BMI-matched recruitment or multivariable logistic regression to estimate the independent genetic effect.

Family history was more common among women with PCOS, but the DD comparison in the familial subgroup did not remain significant after multiplicity correction. Familial aggregation is consistent with the complex genetic architecture of PCOS, yet self-reported history is an imprecise proxy for inherited susceptibility because relatives’ diagnoses were not verified and shared environmental factors cannot be separated from genetic effects. The family-history analysis should therefore be viewed as exploratory rather than evidence that rs35569394 explains familial PCOS.

Taken together, the results identify rs35569394 as a candidate marker warranting further investigation in Turkish women rather than as a diagnostic or predictive test. A clinically useful marker would require reproducible effects across independent cohorts, stability after adjustment for adiposity and ancestry, consistency across Rotterdam phenotypes, and evidence linking genotype to VEGFA expression or ovarian function. The present study provides an initial population-specific signal but does not meet those requirements for clinical translation.

### Limitations

The main limitations were the control-group HWE deviation, incomplete BMI matching without an available covariate-adjusted model, domain-based rather than global multiplicity correction, and limited power for subgroup and multiplicity-adjusted analyses. These factors increase the possibility of genotyping, selection, confounding, or type I/II error despite repeat-genotyping concordance.

Rotterdam A–D phenotypes could not be reconstructed reliably, family history was self-reported, and no VEGFA expression or functional assays were performed. The pooled association should therefore be considered preliminary and not extrapolated to a particular phenotype or to clinical utility^24,25^. Larger, well-matched studies with prespecified quality control, ancestry assessment, phenotype stratification, unified multiplicity correction, and functional validation are needed.

## CONCLUSION

In this Turkish case-control sample, the VEGFA −2549 I/D DD genotype was more frequent among women with PCOS than among controls. This study shows an observed association; it does not establish causality, functional effects, phenotype-specific relevance, or clinical utility. Control-group HWE deviation, residual confounding from incomplete matching, limited power, non-global multiplicity correction, absence of phenotype stratification, and lack of functional validation make the finding preliminary. Independent replication in larger, well-matched cohorts with prespecified genotyping quality control, ancestry assessment, covariate analysis, phenotype stratification, and functional studies is required.

## Data Availability

The datasets generated and/or analyzed during the current study are available from the corresponding author upon reasonable request.
